# Early Surgical Intervention in a Case of Primary Cardiac Lymphoma in an Immunocompetent 56-Year-Old Male

**DOI:** 10.7759/cureus.111139

**Published:** 2026-06-19

**Authors:** Joel Diaz-Portorreal, Sahil Verma, Robert Bender, Samantha Arzillo, Alfredo Rego

**Affiliations:** 1 Department of Anesthesiology, HCA Florida Westside Hospital, Plantation, USA; 2 Department of Cardiothoracic Surgery, HCA Florida Westside Hospital, Plantation, USA; 3 Department of Pathology, HCA Florida Westside Hospital, Plantation, USA

**Keywords:** cardiac mass resection, cardiothoracic and vascular surgery, cardiothoracic anesthesia, histopathology, immunocompetent patients, intra-cardiac mass, large b-cell lymphomas, non-hodgkin’s lymphomas, primary cardiac lymphoma (pcl), transesophageal echo

## Abstract

Primary cardiac lymphomas are rare, high-mortality malignancies requiring a rapid, multidisciplinary response for effective management. We present the case of a 56-year-old immunocompetent male with a three-day history of intermittent chest pain, abdominal pain, visual disturbances, and headaches, leading to the identification of a right-sided cardiac mass. The patient underwent urgent surgical exploration and excision of the mass for symptomatic relief and was subsequently referred for adjuvant chemotherapy.

## Introduction

Primary cardiac lymphomas (PCLs) are rare, accounting for fewer than 1-2% of primary cardiac tumors and 0.5% of extranodal non-Hodgkin’s lymphomas [1]. Presentation of PCL mimics that of many other cardiopulmonary pathologies such as dyspnea, arrhythmia, and pericardial effusion, with some cases reporting superior vena cava syndrome as a rare symptom [2]. Risk factors for PCL include immunocompromised and HIV-positive patients, with a minority of patients immunocompetent at the time of diagnosis [3,4]. In a study of 306 patients identified between 2004 and 2016, the majority of patients were white (82%), male (54.5%), and 68.7 ± 14.2 years of age [5]. Chemotherapy remains the mainstay of treatment for patients presenting with PCL [2]. Unfortunately, the general prognosis of PCL is poor, with a median survival of five years after treatment [3,5,6].

## Case presentation

A 56-year-old Hispanic male with a history of gastroesophageal reflux disease, polysubstance abuse, and 10 pack-years of tobacco use presented to the emergency department with a three-day history of waxing and waning substernal chest pain radiating to bilateral arms, worsening with inspiration. Additionally, he reported a one-month history of 8/10 intermittent epigastric pain associated with food intolerance and nausea, as well as visual disturbances and headaches.

Upon examination, vital signs were temperature of 97.9°F, heart rate of 120 beats/minute, blood pressure of 150/101 mmHg, and oxygen saturation of 98% on room air. The patient appeared uncomfortable but in no acute distress. Cardiac examination revealed S1S2 regular rate and rhythm, a 3/6 murmur, and no jugular venous distension. Lungs were clear, and the abdominal examination revealed tenderness in the right upper quadrant without guarding or rebound tenderness.

Laboratory results are summarized in Table 1 and showed leukocytosis, mild anemia, thrombocytopenia, and transaminitis. Presenting EKG demonstrated age-indeterminate inferior and anterior infarcts with prolonged QT intervals (Figure 1). An echocardiogram revealed an ejection fraction of 30%, diastolic dysfunction, wall motion abnormalities, and a 5.8 cm mobile mass in the lateral right atrium (Figure 2). Right upper quadrant abdominal ultrasound imaging showed findings suggestive of acalculous cholecystitis (Figure 3). This finding was further explored with a CT scan of the abdomen and pelvis with contrast, which did not find any acute abdominopelvic processes; however, a CT scan of the chest with contrast revealed a right-sided heart lesion (Figures 4, 5).

**Table 1:** Summary of select blood laboratory results. AST = aspartate aminotransferase; ALT = alanine aminotransferase

Diagnostic laboratory test	Value	Reference range
White blood cell count	11.9 × 10^3^/µL	4.0–10.5 × 10^3^/µL
Hemoglobin	13.1 g/dL	13.7–17.5 g/dL
Hematocrit	39.3%	40.1–51.0%
Platelet count	136 × 10^3^/µL	150–400 × 10^3^/µL
Sodium level	141 mmol/L	135–145 mmol/L
Potassium level	3.4 mmol/L	3.5–5.2 mmol/L
Creatinine	0.98 mg/dL	0.43–1.13 mg/dL
AST	55 U/L	10–40 U/L
ALT	119 U/L	10–60 U/L
Troponin	0.012 ng/mL	0.000–0.034 ng/mL

**Figure 1:**
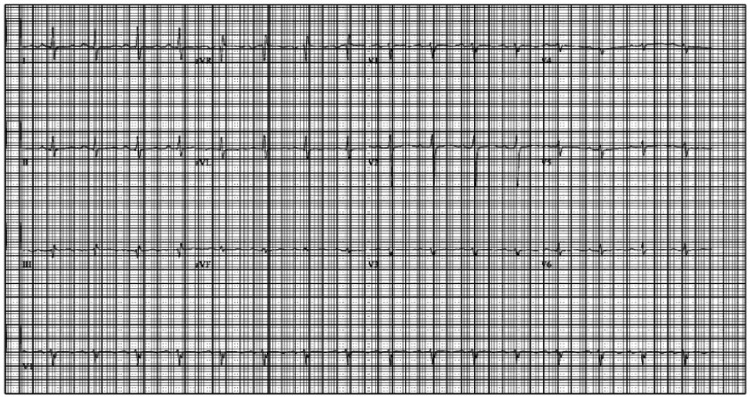
Preoperative 12-lead EKG.

**Figure 2:**
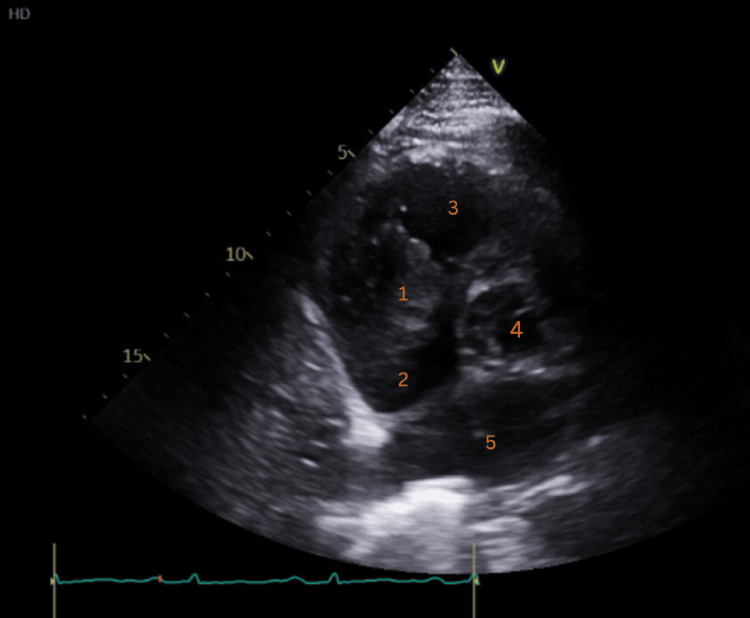
Preoperative transthoracic echocardiogram in parasternal short-axis view at the level of the aortic valve. The orange numbered labels represent the following structures: right atrial mass (1), right atrium (2), right ventricular outflow (3), aortic valve in short axis (4), and the left atrium (5).

**Figure 3:**
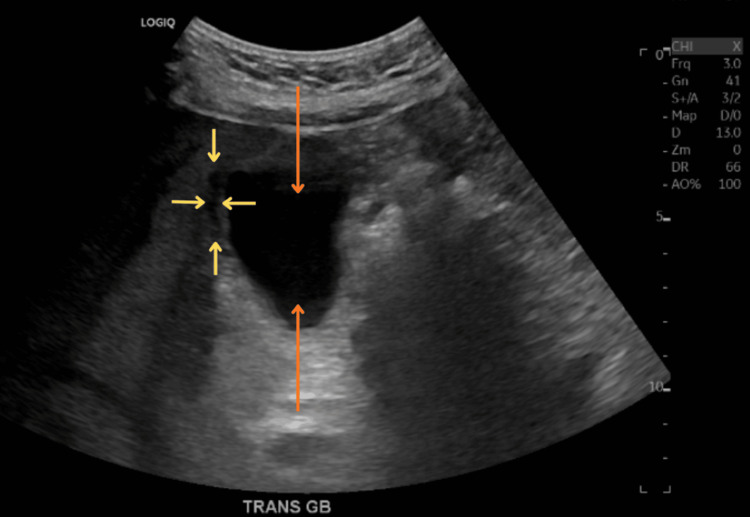
Right upper quadrant abdominal ultrasound in transverse axis, performed preoperatively. The orange arrows are pointing to the gallbladder in the center of the image. Yellow arrows are found outside of the gallbladder wall pointing to an anechoic focus that is concerning for pericholecystic fluid.

**Figure 4:**
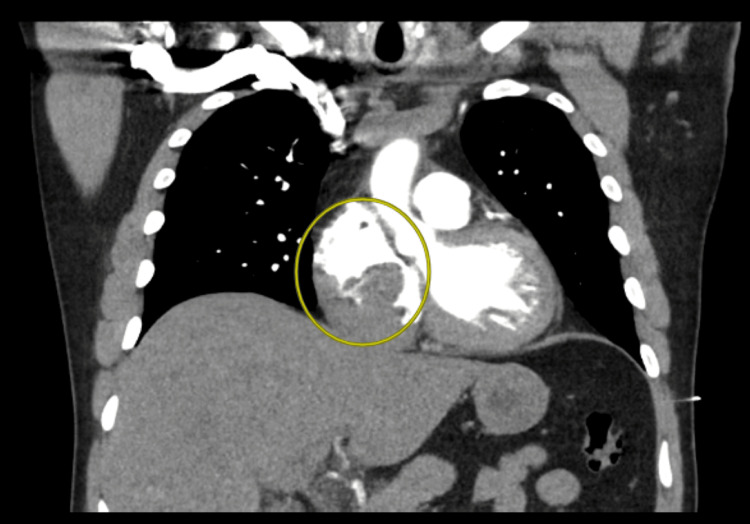
CT of the chest with contrast (coronal view) displaying the 5.8 cm right atrial mass, circled in yellow.

**Figure 5:**
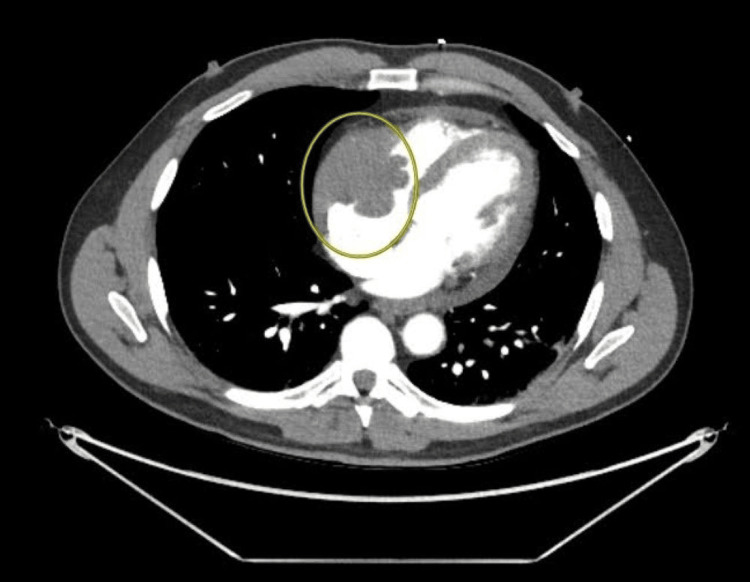
CT of the chest with contrast (axial view) displaying the 5.8 cm right atrial mass, circled in yellow.

The patient was admitted for further evaluation. Duplex carotid ultrasound showed no stenosis, and cardiac catheterization revealed patent coronary vessels without stenosis. A preoperative transesophageal echocardiogram re-identified the mass. A brain CT ruled out acute intracranial abnormalities. The decision was made to take the patient for surgery, which occurred on the seventh day of admission after his preoperative workup was completed.

For the procedure, a pre-induction arterial line was placed, and adequate intravenous access was established. The patient was taken to the operating room and had all other standard monitors placed. After adequate preoxygenation, induction of anesthesia occurred with administration of midazolam, fentanyl, etomidate, succinylcholine, and esmolol. The patient was intubated with an oral endotracheal tube. Maintenance of anesthesia was established with sevoflurane and rocuronium. A right internal jugular central venous catheter was placed; a pulmonary artery catheter was not inserted given the location of the mass and the planned surgical site. Additional monitors were placed, including cerebral oximetry and hemodynamic monitoring (e.g., continuous cardiac output, cardiac index, stroke volume variation). For postoperative pain control, bilateral parasternal nerve blocks were performed.

Before the start of the surgery, transesophageal echocardiography (TEE) was performed, with the mass being identified (Figures 6, 7). Of note, the mass was located in the lateral wall of the right atrium, extending from the atrium through the tricuspid valve into the right ventricle, measuring 5.27 cm × 5.19 cm. The patient was noted to have a dilated right atrium and severe tricuspid stenosis as a result of the mass, with a mean gradient of 10 mmHg. Other findings on TEE included mildly reduced biventricular systolic function and mild global hypokinesis.

**Figure 6:**
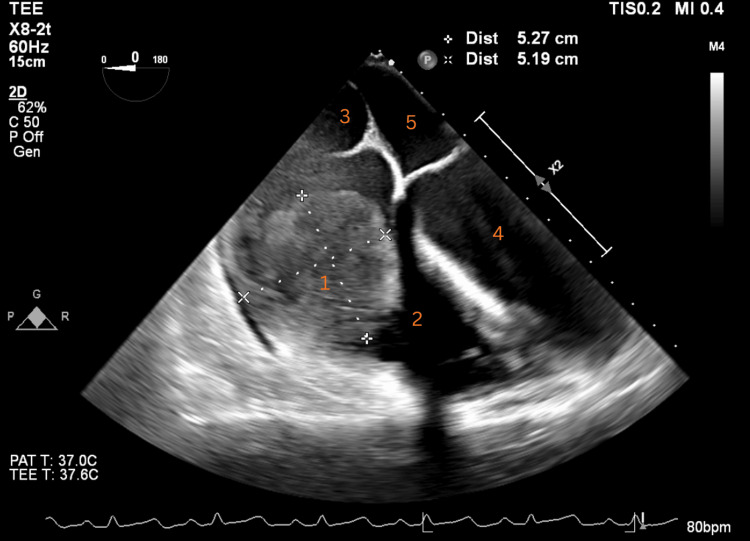
Intraoperative transesophageal echocardiography with the mid-esophageal four-chamber view demonstrating the right atrial mass. The orange numbered labels are as follows: mass measurement at 5.27 cm × 5.19 cm (1), right ventricle (2), right atrium (3), left ventricle (4), and left atrium (5).

**Figure 7:**
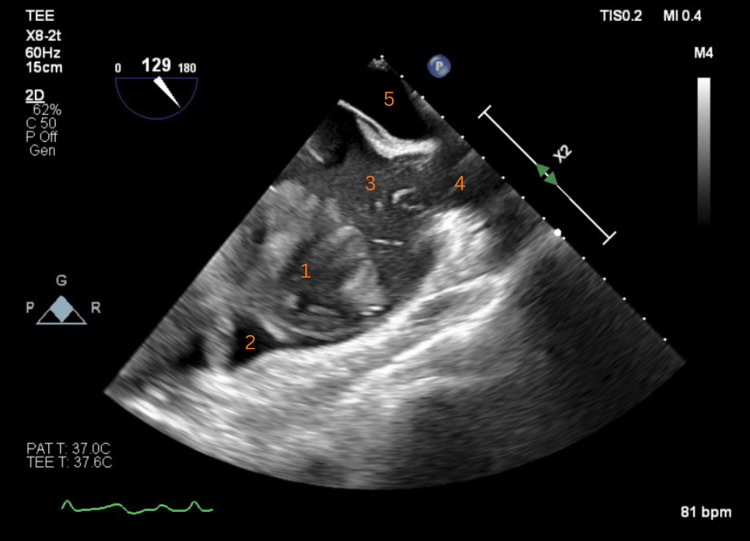
Mid-esophageal modified bicaval view on transesophageal echocardiography. The orange numbered labels are as follows: right-sided cardiac mass (1), right ventricle inflow (2), right atrium (3), superior vena cava (4), and left atrium (5).

The operative portion of the procedure then followed with uneventful establishment of cardiopulmonary bypass. The patient then underwent surgical exploration and removal of the right atrial mass, with septum, annulus, wall of the right leaflet, and the conduction system debulking. Intraoperative images of the mass in situ and after resection are shown in Figure 8 and Figure 9, respectively. The tricuspid valve was replaced with a 27 mm bioprosthetic due to tumor involvement. Coming off of cardiopulmonary bypass occurred without issue. Post-bypass TEE was performed and showed interval removal of the mass with the new bioprosthetic valve well seated. There was no tricuspid stenosis with a mean gradient of 2 mmHg, an improvement from prior. No tricuspid regurgitation was identified. Overall, there was a noted improvement in biventricular systolic function post-procedure. The patient remained on low-dose infusions of epinephrine and norepinephrine coming off of bypass, which continued through transfer of the patient to the intensive care unit.

**Figure 8:**
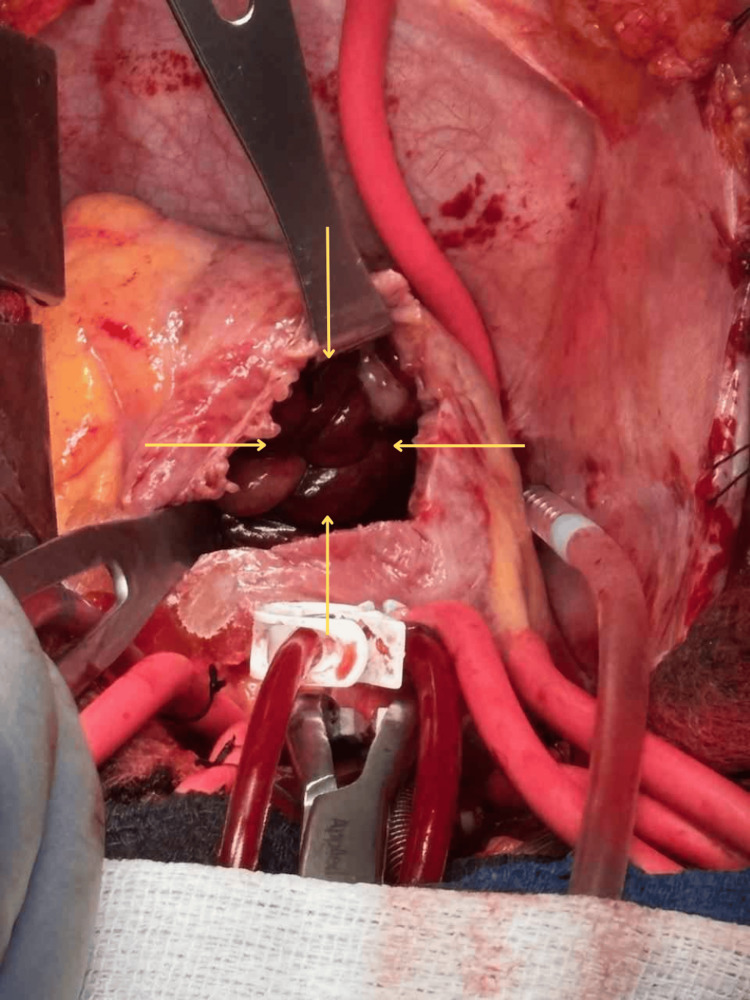
Operative view in the surgical field, with yellow arrows pointing to the right atrial mass.

**Figure 9:**
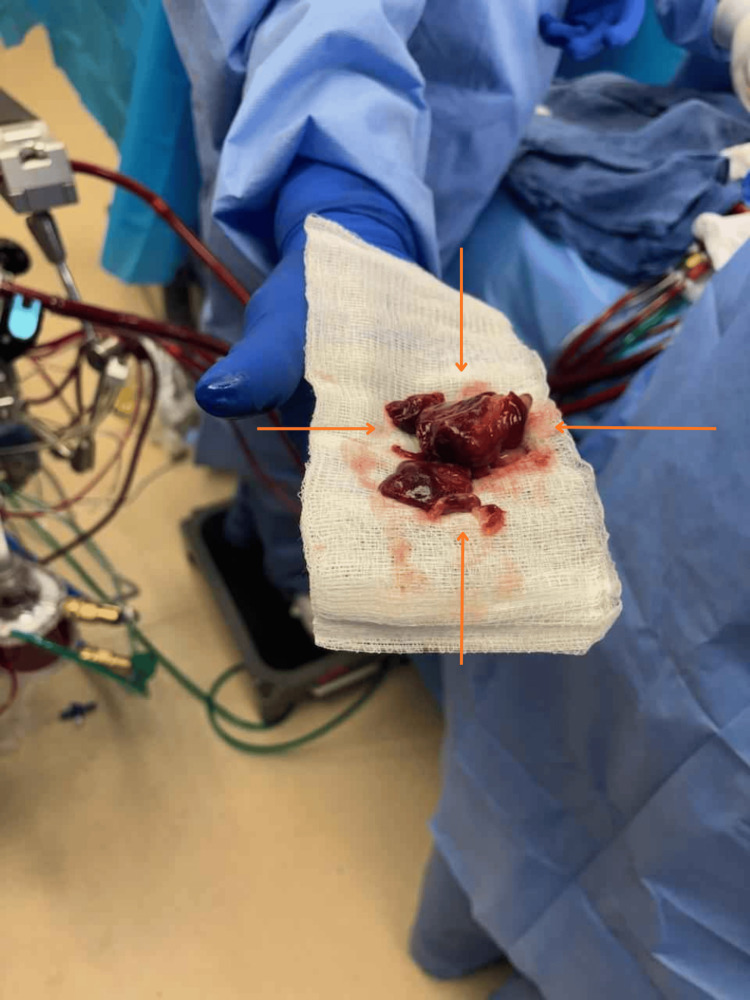
Mass displayed on a surgical sponge, with orange arrows pointing to the right atrial mass after resection.

Pathology confirmed large B-cell non-Hodgkin’s lymphoma with aggressive features. Low-power and high-power histopathological images of the cardiac mass can be found in Figure 10 and Figure 11, respectively. Extensive areas of sampling from the mass showed sheets of malignant tumor cells that infiltrate cardiac muscle bundles and areas of marked necrosis. The sheets of malignant tumor cells also demonstrated a starry-sky appearance with marked apoptosis, mitotic figures, and cells that were large and showed visible nucleoli.

**Figure 10:**
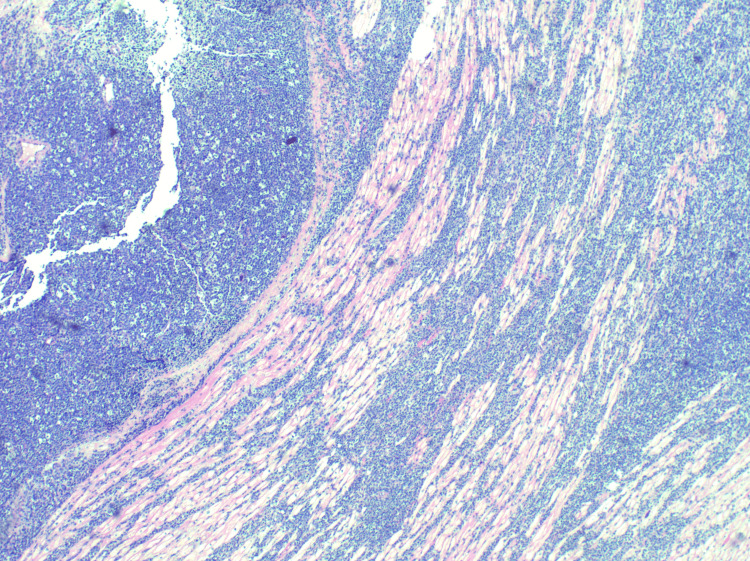
Low-power histopathological image of the resected right atrial mass.

**Figure 11:**
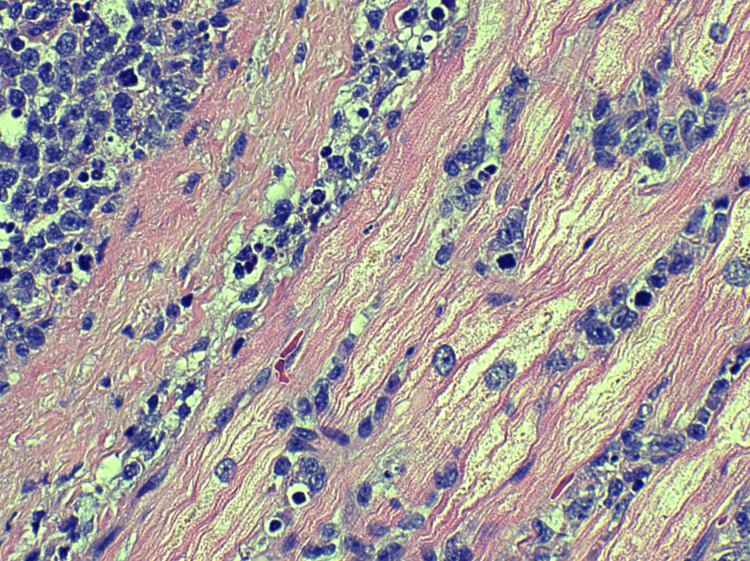
High-power histopathological image of the resected right atrial mass.

The fluorescence in situ hybridization analysis showed positivity for BCL-6 rearrangement in 89% of the cells analyzed. Flow cytometric analysis revealed a monoclonal B-cell population expressing CD45, CD19, and CD20, indicating lambda light chain restriction. Immunohistochemical staining with Ki-67 showed a proliferative index of 80-90%. Biopsies of the right atrial wall, papillary muscle, and tricuspid valve were consistent with the above diagnosis.

Postoperatively, the patient experienced bradycardia, which was worked up and identified as a complete heart block, as diagnosed with EKG (Figure 12), necessitating temporary pacing through intraoperatively placed pacer wires that were still present. A pacemaker was implanted on postoperative day five, and a chemotherapy port was placed on day six. A bone marrow biopsy was performed before discharge in which no morphological or immunophenotypic evidence of acute leukemia or B-cell lymphoma was seen. The patient was discharged on postoperative day eight and scheduled for outpatient chemotherapy. Due to pacemaker insertion, MRI would only be possible on postoperative week six.

**Figure 12:**
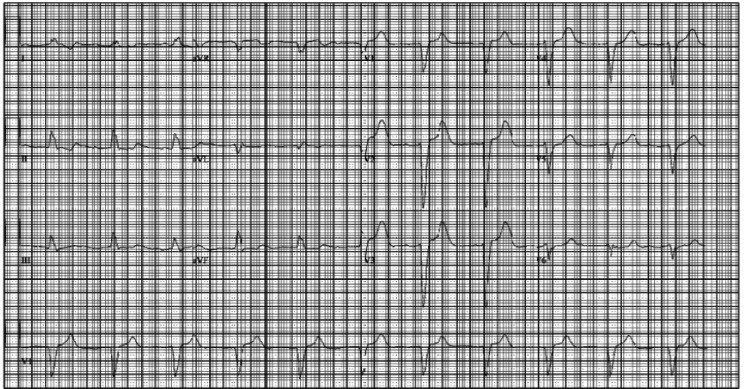
Postoperative 12-lead EKG with conduction system changes from baseline EKG showing complete heart block.

## Discussion

PCL is a rare and highly lethal cardiac tumor that is primarily treated with chemotherapy; however, precise surgical dissection and excellent intraoperative skills are essential tools for symptomatic management. As with all cardiac tumors, ample attention to the patient’s presenting symptoms, signs, and laboratory values may facilitate early identification and diagnosis of these tumors. A patient such as ours who presented with embolic signs of vision changes and headaches should immediately prompt imaging and cardiac workup, which includes a CT chest with contrast, echocardiography, and EKG. It is possible and reasonable that many patients may present with this as an incidental finding early on, and this may be discovered incidentally during evaluation for another suspected process; this should not delay urgent evaluation, as embolic events may rapidly become life-threatening.

It is important to keep a broad differential early on, as common keywords and phrases such as “right atrial mass” can make physicians consider options such as myxomas [7] or lipomas [8], which are benign etiologies. Some facilities employ use of transcatheter echocardiogram biopsies for early identification of these cardiac masses [9]. This is highly region and patient-dependent, with our facility choosing to employ surgical intervention and intraoperative biopsies as our patient was presently symptomatic with chest pain. Attention to the patient’s past medical history and complete review of systems can also provide clues as to whether this disease process is primary or secondary in nature. PCLs tend to present without B-symptoms of fevers, weight loss, and night sweats, and may not present with pericardial tamponade. On the contrary, secondary cardiac lymphomas will have B-symptoms followed by cardiac involvement, with the final pathology found for many at autopsy [10]. These secondary cardiac lymphomas are associated with a worse prognosis [11,12], and are more common causes of cardiac lymphoma.

Management of PCLs is R-CHOP (rituximab, cyclophosphamide, hydroxydaunorubicin, oncovin, prednisone)-based chemotherapy, which has been associated with favorable response rates [2,13]. Chemotherapy does come with its own risks; these risks are numerous, well-studied, and must be discussed with the patient and their goals of care in the context of a recently diagnosed cardiac malignancy. The median time for survival for many of these patients without treatment is around one month; treatment can extend the median time of survival to around five years [5]. Notably, surgical, radiation, and immune-based treatments do not appear to have a significant effect on long-term outcomes in comparison to chemotherapy. These approaches should not be completely discarded, as de-bulking followed by chemotherapy may improve the response rate to treatment [14].

Anesthetic management and goals for resection of cardiac lymphoma are similar to the resection of other intracardiac tumors. There is the potential for cardiovascular instability/collapse caused by obstruction of venous return by the mass, as well as limitations in normal flow through the heart [15]. These findings can be manifested as hemodynamic variations/decompensation [15] during intraoperative periods such as induction. For this reason, agents that cause expected decreases in systemic vascular resistance were avoided for induction, and vasoactive agents were available in case the patient became unstable. Other considerations for a case of this nature are fragmentation of the tumor, not only during mass resection but with other procedures such as central venous catheter placement [15,16]. For this reason, the use of TEE was essential to this procedure as with most cardiac procedures. It allowed for guidance of line placement, mass characterization, surgical planning, and preoperative/postoperative echocardiographic indices. Close postoperative neurologic monitoring of the patient is indicated given the potential for a fragment of the mass to embolize and cause a cerebrovascular accident. Lastly, given the proximity of the mass to the conduction system, development of arrhythmias postoperatively is fairly common, with permanent pacing as an anticipated outcome [16].

Frequent and close follow-up should be done for patients undergoing surgical debulking and those undergoing chemotherapy, considering the rapidly mortal nature of this disease.

## Conclusions

This case report describes the presentation of a 56-year-old male who underwent surgical resection of a right-sided intracardiac tumor, namely, PCL. Intraoperatively, the patient underwent a surgical resection of the right atrial mass, with septum, annulus, wall of the right leaflet, and the conduction system debulking. Postoperatively, the patient was managed in the intensive care unit, with the only major complication being the development of complete heart block with subsequent pacemaker insertion. He was otherwise managed medically and eventually discharged home with subsequent initiation of chemotherapy. This report highlights the importance of urgent surgical intervention to alleviate symptoms in patients with PCL, providing a bridge to chemotherapy to address the underlying malignancy. This case underscores the importance of timely diagnosis and a multidisciplinary approach in managing rare cardiac tumors.
